# (*E*)-1-(4-Fluoro­phen­yl)-3-(4-methyl­phen­yl)prop-2-en-1-one

**DOI:** 10.1107/S1600536808011483

**Published:** 2008-04-30

**Authors:** Hoong-Kun Fun, Samuel Robinson Jebas, P. S. Patil, E. Deepak D’Silva, S. M. Dharmaprakash

**Affiliations:** a X-ray Crystallography Unit, School of Physics, Universiti Sains Malaysia, 11800 USM, Penang, Malaysia; b Department of Studies in Physics, Mangalore University, Mangalagangotri, Mangalore 574 199, India

## Abstract

The title compound, C_16_H_13_FO, adopts an *E* configuration with respect to the C=C bond of the propenone unit. The dihedral angle between the two benzene rings is 47.0 (5)°. Intra­molecular C—H⋯O hydrogen bonds generate an *S*(5) ring motif. In the crystal structure, mol­ecules are packed into columns along the *c* axis and the structure is stabilized by weak intra­molecular C—H⋯O hydrogen bonds and inter­molecular C—H⋯π inter­actions involving both aromatic rings.

## Related literature

For applications of chalcones in non-linear optics, see, for example, Agrinskaya *et al.* (1999 ▶); Gu *et al.* (2008 ▶); Patil *et al.* (2007*a* ▶,*b* ▶,*c* ▶). For related structures see: Patil *et al.* (2007*a* ▶,*b* ▶,*c* ▶). For graph-set analysis of hydrogen bonding, see: Bernstein *et al.* (1995 ▶).
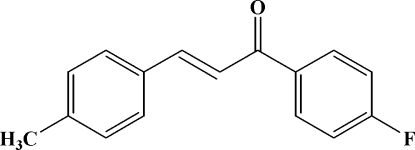


## Experimental

### 

#### Crystal data

C_16_H_13_FO
*M* *_r_* = 240.93
Monoclinic, 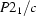

*a* = 14.505 (2) Å
*b* = 14.0523 (18) Å
*c* = 5.8382 (8) Å
β = 92.042 (10)°
*V* = 1189.3 (3) Å^3^
*Z* = 4
Mo *K*α radiation
μ = 0.09 mm^−1^
*T* = 100.0 (1) K
0.47 × 0.15 × 0.07 mm

#### Data collection

Bruker SMART APEXII CCD area-detector diffractometer
Absorption correction: multi-scan (*SADABS*; Bruker, 2005 ▶) *T*_min_ = 0.913, *T*_max_ = 0.993
14807 measured reflections
3442 independent reflections
2256 reflections with *I* > 2σ(*I*)
*R*_int_ = 0.050

#### Refinement

*R*[*F* ^2^ > 2σ(*F* ^2^)] = 0.055
*wR*(*F* ^2^) = 0.141
*S* = 1.07
3442 reflections
164 parameters
H-atom parameters constrained
Δρ_max_ = 0.36 e Å^−3^
Δρ_min_ = −0.26 e Å^−3^

### 

Data collection: *APEX2* (Bruker, 2005 ▶); cell refinement: *APEX2*; data reduction: *SAINT* (Bruker, 2005 ▶); program(s) used to solve structure: *SHELXTL* (Sheldrick, 2008 ▶); program(s) used to refine structure: *SHELXTL*; molecular graphics: *SHELXTL* (Sheldrick, 2008 ▶); software used to prepare material for publication: *SHELXTL* and *PLATON* (Spek, 2003 ▶).

## Supplementary Material

Crystal structure: contains datablocks global, I. DOI: 10.1107/S1600536808011483/sj2485sup1.cif

Structure factors: contains datablocks I. DOI: 10.1107/S1600536808011483/sj2485Isup2.hkl

Additional supplementary materials:  crystallographic information; 3D view; checkCIF report

## Figures and Tables

**Table 1:** Hydrogen-bond geometry (Å, °)

*D*—H⋯*A*	*D*—H	H⋯*A*	*D*⋯*A*	*D*—H⋯*A*
C9—H9*A*⋯O1	0.93	2.50	2.820 (2)	100
C5—H5*A*⋯*Cg*1^i^	0.93	2.96	3.525	120
C9—H9*A*⋯*Cg*1^ii^	0.93	3.02	3.604	123
C2—H2*A*⋯*Cg*2^iii^	0.93	3.01	3.635	126
C14—H14*A*⋯*Cg*2^iv^	0.93	2.76	3.452	132

Symmetry codes: (i) 
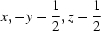
; (ii) 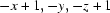; (iii) 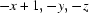; (iv) 
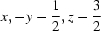
. *Cg*1 is the centroid of the ring C1–C6 and *Cg*2 is the centroid of the ring C10–C15.

**Fig. 1.**
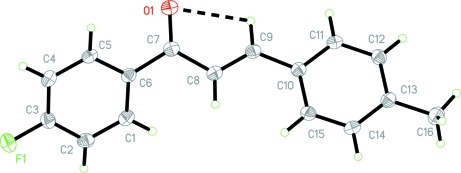
The asymmetric unit of the title compound, showing 50% probability displacement ellipsoids and the atom numbering scheme. The intramolecular H-bond is drawn as a dashed line.

**Fig. 2.**
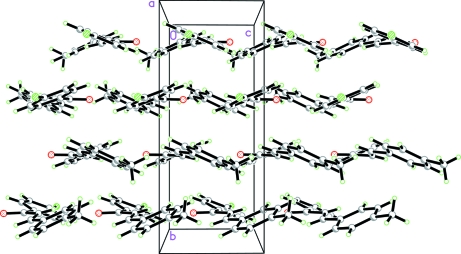
The crystal packing of the title compound, viewed along the a axis.

## supplementary crystallographic information

### Comment

Significant studies on applications of chalcones in nonlinear optics have motivated us to further to identify materials, especially chalcone derivatives, with appropriate absorption in the UV region along with a transmission window in the NIR range contributing to multi-photon absorption (Agrinskaya *et al.*, 1999; Gu *et al.*, 2008; Patil *et al.*, 2007a-c). Here we report the crystal structure of the title chalcone derivative, (I), Fig. 1.

In (I), the molecule exhibits an E configuration with respect to the C8═C9 double bond with the C7–C8–C9–C10 torsion angle 174.6 (2)°. The bond lengths and angles in (I) are comparable to those observed in related structures (Patil *et al.*, 2007a-c). The dihedral angle between the two benzene rings is 47.0 (2)°.

Intramolecular C—H···O hydrogen bonds generate an S(5) ring motif. In the crystal structure molecules are packed into columns along the *c* axis and the structure is stabilised by weak intramolecular C—H···O hydrogen bonds and intermolecular C—H···π interactions involving both aromatic rings, Table 1.

### Experimental

The compound (I) was synthesized by the condensation of p-tolualdehyde (0.01 mol) with 4-fluoroacetophenone (0.01 mol) in methanol (60 ml) in the presence of a catalytic amount of sodium hydroxide solution (5 ml, 30%). After stirring (4 h), the contents of the flask were poured into ice-cold water (500 ml) and left to stand for 5 h. The resulting crude solid was filtered and dried. The precipitated compound was recrystallized from acetone.

### Refinement

All the H atoms were positioned geometrically and refined using a riding model with C–H = 0.93Å for aromatic and 0.96Å for CH_3_. The *U*_iso_ values were constrained to be 1.5*U*_eq_ of the carrier atom for the methyl H atoms and 1.2U_equ_ for the remaining hydrogen atoms.

### Crystal data

**Table:**

C_16_H_13_FO	*F*_000_ = 504
*M**_r_* = 240.93	*D*_x_ = 1.342 Mg m^−^^3^
Monoclinic, *P*2_1_/*c*	Mo *K*α radiation λ = 0.71073 Å
Hall symbol: -P 2ybc	Cell parameters from 1943 reflections
*a* = 14.505 (2) Å	θ = 2.8–34.6º
*b* = 14.0523 (18) Å	µ = 0.09 mm^−^^1^
*c* = 5.8382 (8) Å	*T* = 100.0 (1) K
β = 92.042 (10)º	Plate, colourless
*V* = 1189.3 (3) Å^3^	0.47 × 0.15 × 0.07 mm
*Z* = 4	

### Data collection

**Table:**

Bruker SMART APEXII CCD area-detector diffractometer	3442 independent reflections
Radiation source: fine-focus sealed tube	2256 reflections with *I* > 2σ(*I*)
Monochromator: graphite	*R*_int_ = 0.050
*T* = 100.0(1) K	θ_max_ = 30.0º
φ and ω scans	θ_min_ = 2.0º
Absorption correction: multi-scan(SADABS; Bruker, 2005)	*h* = −20→16
*T*_min_ = 0.913, *T*_max_ = 0.993	*k* = −19→15
14807 measured reflections	*l* = −8→8

### Refinement

**Table:**

Refinement on *F*^2^	Secondary atom site location: difference Fourier map
Least-squares matrix: full	Hydrogen site location: inferred from neighbouring sites
*R*[*F*^2^ > 2σ(*F*^2^)] = 0.055	H-atom parameters constrained
*wR*(*F*^2^) = 0.141	*w* = 1/[σ^2^(*F*_o_^2^) + (0.0591*P*)^2^ + 0.1964*P*] where *P* = (*F*_o_^2^ + 2*F*_c_^2^)/3
*S* = 1.07	(Δ/σ)_max_ < 0.001
3442 reflections	Δρ_max_ = 0.36 e Å^−^^3^
164 parameters	Δρ_min_ = −0.26 e Å^−^^3^
Primary atom site location: structure-invariant direct methods	Extinction correction: none

### Special details

**Table:**

Experimental. The data was collected with the Oxford Cyrosystem Cobra low-temperature attachment.
Geometry. All e.s.d.'s (except the e.s.d. in the dihedral angle between two l.s. planes) are estimated using the full covariance matrix. The cell e.s.d.'s are taken into account individually in the estimation of e.s.d.'s in distances, angles and torsion angles; correlations between e.s.d.'s in cell parameters are only used when they are defined by crystal symmetry. An approximate (isotropic) treatment of cell e.s.d.'s is used for estimating e.s.d.'s involving l.s. planes.
Refinement. Refinement of *F*^2^ against ALL reflections. The weighted *R*-factor *wR* and goodness of fit *S* are based on *F*^2^, conventional *R*-factors *R* are based on *F*, with *F* set to zero for negative *F*^2^. The threshold expression of *F*^2^ > σ(*F*^2^) is used only for calculating *R*-factors(gt) *etc*. and is not relevant to the choice of reflections for refinement. *R*-factors based on *F*^2^ are statistically about twice as large as those based on *F*, and *R*- factors based on ALL data will be even larger.

### Fractional atomic coordinates and isotropic or equivalent isotropic displacement parameters (Å^2^)

**Table:**

	*x*	*y*	*z*	*U*_iso_*/*U*_eq_	
F1	0.13263 (6)	0.62202 (8)	0.22333 (18)	0.0335 (3)	
O1	0.52448 (8)	0.62178 (9)	−0.19348 (19)	0.0299 (3)	
C1	0.37893 (11)	0.59097 (11)	0.2869 (3)	0.0223 (3)	
H1A	0.4227	0.5697	0.3955	0.027*	
C2	0.28643 (11)	0.58906 (11)	0.3360 (3)	0.0226 (4)	
H2A	0.2671	0.5657	0.4754	0.027*	
C3	0.22364 (11)	0.62262 (11)	0.1734 (3)	0.0225 (4)	
C4	0.24829 (11)	0.65754 (11)	−0.0361 (3)	0.0235 (4)	
H4A	0.2041	0.6804	−0.1415	0.028*	
C5	0.34102 (11)	0.65749 (11)	−0.0847 (3)	0.0211 (3)	
H5A	0.3594	0.6797	−0.2260	0.025*	
C6	0.40712 (10)	0.62458 (11)	0.0752 (3)	0.0193 (3)	
C7	0.50568 (11)	0.62320 (11)	0.0105 (3)	0.0220 (3)	
C8	0.57812 (11)	0.62535 (11)	0.1937 (3)	0.0229 (3)	
H8A	0.5628	0.6413	0.3423	0.027*	
C9	0.66551 (11)	0.60475 (11)	0.1492 (3)	0.0203 (3)	
H9A	0.6764	0.5840	0.0014	0.024*	
C10	0.74563 (10)	0.61113 (10)	0.3059 (3)	0.0192 (3)	
C11	0.83138 (10)	0.57998 (11)	0.2337 (3)	0.0202 (3)	
H11A	0.8354	0.5513	0.0908	0.024*	
C12	0.91061 (11)	0.59096 (11)	0.3709 (3)	0.0216 (3)	
H12A	0.9668	0.5695	0.3189	0.026*	
C13	0.90722 (11)	0.63365 (11)	0.5856 (3)	0.0211 (3)	
C14	0.82142 (11)	0.66329 (11)	0.6599 (3)	0.0210 (3)	
H14A	0.8176	0.6913	0.8037	0.025*	
C15	0.74201 (11)	0.65200 (11)	0.5248 (3)	0.0207 (3)	
H15A	0.6856	0.6717	0.5795	0.025*	
C16	0.99320 (12)	0.64989 (13)	0.7314 (3)	0.0290 (4)	
H16A	1.0451	0.6225	0.6577	0.044*	
H16B	0.9866	0.6205	0.8784	0.044*	
H16C	1.0029	0.7170	0.7512	0.044*	

### Atomic displacement parameters (Å^2^)

**Table:**

	*U*^11^	*U*^22^	*U*^33^	*U*^12^	*U*^13^	*U*^23^
F1	0.0209 (5)	0.0457 (7)	0.0340 (6)	−0.0022 (4)	0.0031 (4)	0.0022 (5)
O1	0.0265 (6)	0.0434 (8)	0.0196 (6)	0.0016 (5)	0.0013 (5)	−0.0004 (5)
C1	0.0258 (8)	0.0201 (8)	0.0207 (8)	0.0015 (6)	−0.0029 (6)	−0.0004 (6)
C2	0.0279 (8)	0.0209 (8)	0.0192 (8)	−0.0029 (6)	0.0017 (6)	0.0011 (6)
C3	0.0198 (8)	0.0213 (8)	0.0264 (9)	−0.0011 (6)	0.0016 (6)	−0.0017 (7)
C4	0.0240 (8)	0.0235 (8)	0.0226 (8)	−0.0001 (6)	−0.0044 (6)	0.0007 (7)
C5	0.0256 (8)	0.0215 (8)	0.0160 (8)	−0.0026 (6)	−0.0013 (6)	0.0001 (6)
C6	0.0216 (8)	0.0178 (8)	0.0184 (8)	0.0012 (6)	−0.0011 (6)	−0.0024 (6)
C7	0.0243 (8)	0.0213 (8)	0.0203 (8)	−0.0003 (6)	0.0006 (6)	−0.0005 (7)
C8	0.0241 (8)	0.0259 (9)	0.0185 (8)	0.0001 (6)	−0.0007 (6)	−0.0017 (7)
C9	0.0241 (8)	0.0189 (8)	0.0180 (8)	−0.0015 (6)	0.0007 (6)	0.0005 (6)
C10	0.0218 (8)	0.0171 (8)	0.0185 (8)	−0.0016 (6)	0.0005 (6)	0.0023 (6)
C11	0.0246 (8)	0.0199 (8)	0.0160 (8)	−0.0009 (6)	0.0012 (6)	−0.0002 (6)
C12	0.0204 (8)	0.0211 (8)	0.0235 (8)	0.0026 (6)	0.0017 (6)	0.0003 (6)
C13	0.0233 (8)	0.0195 (8)	0.0203 (8)	−0.0006 (6)	−0.0017 (6)	0.0020 (6)
C14	0.0274 (8)	0.0192 (8)	0.0163 (8)	0.0002 (6)	−0.0001 (6)	0.0000 (6)
C15	0.0217 (8)	0.0204 (8)	0.0202 (8)	0.0009 (6)	0.0029 (6)	0.0013 (6)
C16	0.0277 (9)	0.0317 (10)	0.0273 (9)	0.0009 (7)	−0.0041 (7)	−0.0021 (7)

### Geometric parameters (Å, °)

**Table:**

F1—C3	1.3621 (17)	C9—C10	1.456 (2)
O1—C7	1.2315 (18)	C9—H9A	0.9300
C1—C2	1.382 (2)	C10—C11	1.398 (2)
C1—C6	1.398 (2)	C10—C15	1.404 (2)
C1—H1A	0.9300	C11—C12	1.386 (2)
C2—C3	1.375 (2)	C11—H11A	0.9300
C2—H2A	0.9300	C12—C13	1.392 (2)
C3—C4	1.377 (2)	C12—H12A	0.9300
C4—C5	1.385 (2)	C13—C14	1.396 (2)
C4—H4A	0.9300	C13—C16	1.502 (2)
C5—C6	1.393 (2)	C14—C15	1.382 (2)
C5—H5A	0.9300	C14—H14A	0.9300
C6—C7	1.492 (2)	C15—H15A	0.9300
C7—C8	1.473 (2)	C16—H16A	0.9600
C8—C9	1.335 (2)	C16—H16B	0.9600
C8—H8A	0.9300	C16—H16C	0.9600
			
C2—C1—C6	120.43 (15)	C10—C9—H9A	116.3
C2—C1—H1A	119.8	C11—C10—C15	117.72 (14)
C6—C1—H1A	119.8	C11—C10—C9	119.37 (14)
C3—C2—C1	118.31 (14)	C15—C10—C9	122.83 (14)
C3—C2—H2A	120.8	C12—C11—C10	121.27 (14)
C1—C2—H2A	120.8	C12—C11—H11A	119.4
F1—C3—C2	118.24 (14)	C10—C11—H11A	119.4
F1—C3—C4	118.47 (14)	C11—C12—C13	120.90 (14)
C2—C3—C4	123.28 (15)	C11—C12—H12A	119.6
C3—C4—C5	117.83 (15)	C13—C12—H12A	119.6
C3—C4—H4A	121.1	C12—C13—C14	117.96 (15)
C5—C4—H4A	121.1	C12—C13—C16	121.36 (14)
C4—C5—C6	120.87 (14)	C14—C13—C16	120.66 (14)
C4—C5—H5A	119.6	C15—C14—C13	121.50 (15)
C6—C5—H5A	119.6	C15—C14—H14A	119.2
C5—C6—C1	119.26 (14)	C13—C14—H14A	119.2
C5—C6—C7	118.51 (13)	C14—C15—C10	120.61 (14)
C1—C6—C7	122.19 (14)	C14—C15—H15A	119.7
O1—C7—C8	121.73 (14)	C10—C15—H15A	119.7
O1—C7—C6	119.49 (15)	C13—C16—H16A	109.5
C8—C7—C6	118.77 (14)	C13—C16—H16B	109.5
C9—C8—C7	120.80 (15)	H16A—C16—H16B	109.5
C9—C8—H8A	119.6	C13—C16—H16C	109.5
C7—C8—H8A	119.6	H16A—C16—H16C	109.5
C8—C9—C10	127.36 (15)	H16B—C16—H16C	109.5
C8—C9—H9A	116.3		
			
C6—C1—C2—C3	−1.2 (2)	C6—C7—C8—C9	166.14 (15)
C1—C2—C3—F1	−179.03 (13)	C7—C8—C9—C10	174.61 (15)
C1—C2—C3—C4	0.3 (2)	C8—C9—C10—C11	175.44 (15)
F1—C3—C4—C5	−179.85 (13)	C8—C9—C10—C15	−7.9 (3)
C2—C3—C4—C5	0.8 (3)	C15—C10—C11—C12	−1.5 (2)
C3—C4—C5—C6	−1.0 (2)	C9—C10—C11—C12	175.34 (14)
C4—C5—C6—C1	0.2 (2)	C10—C11—C12—C13	−0.1 (2)
C4—C5—C6—C7	178.00 (14)	C11—C12—C13—C14	1.2 (2)
C2—C1—C6—C5	0.9 (2)	C11—C12—C13—C16	−177.22 (15)
C2—C1—C6—C7	−176.76 (15)	C12—C13—C14—C15	−0.8 (2)
C5—C6—C7—O1	−22.4 (2)	C16—C13—C14—C15	177.69 (14)
C1—C6—C7—O1	155.31 (16)	C13—C14—C15—C10	−0.8 (2)
C5—C6—C7—C8	156.49 (15)	C11—C10—C15—C14	1.9 (2)
C1—C6—C7—C8	−25.8 (2)	C9—C10—C15—C14	−174.78 (14)
O1—C7—C8—C9	−15.0 (2)		

### Hydrogen-bond geometry (Å, °)

**Table:**

*D*—H···*A*	*D*—H	H···*A*	*D*···*A*	*D*—H···*A*
C9—H9A···O1	0.93	2.50	2.820 (2)	100
C5—H5A···Cg1^i^	0.93	2.96	3.525	120
C9—H9A···Cg1^ii^	0.93	3.02	3.604	123
C2—H2A···Cg2^iii^	0.93	3.01	3.635	126
C14—H14A···Cg2^iv^	0.93	2.76	3.452	132

Symmetry codes: (i) *x*, −*y*−1/2, *z*−1/2; (ii) −*x*+1, −*y*, −*z*+1; (iii) −*x*+1, −*y*, −*z*; (iv) *x*, −*y*−1/2, *z*−3/2.
